# Supplementary material for: Defective mitochondrial DNA homeostasis in the substantia nigra in Parkinson disease
Source: Nat Commun. 2016 Nov 22;7:13548. doi: 10.1038/ncomms13548 (PMC5121427; doi:10.1038/ncomms13548)
Supplement: Supplementary Information — Supplementary Tables 1-3 [file ncomms13548-s1.pdf]

Subject ID	Gender	Age at onset	Age at death	LB pathology
**PD1**	F	68	81	+
**PD2**	M	72	82	+
**PD3**	M	87	95	+
**PD4**	M	78	86	+
**PD5**	M	77	88	+
**PD6**	M	63	69	+
**PD7**	M	70	82	+
**PD8**	M	63	72	+
**PD9**	F	80	90	+
**PD10**	F	66	78	+
**C1**	M		86	-
**C2**	F		85	-
**C3**	F		81	-
**C4**	M		87	-
**C5**	M		87	-
**C6**	M		79	-
**C7**	M		73	-
**C8**	F		63	-
**C9**	M		63	-
**C10**	M		63	-
**C11**	M		60	-
**C12**	F		59	-
**C13**	M		56	-
**C14**	M		55	-
**C15**	M		52	-
**C16**	F		41	-
**C17**	F		29	NA
**C18**	M		28	NA
**C19**	F		18	NA
**C20**	M		11	NA
**C21**	F		18	NA
**C22**	M		38	NA

Supplementary Table 1. Demographic data and brain pathology of individuals with PD and controls. PD: Parkinson disease; C: control individual; F: female; M: male; NA: not available.

Patient ID	Drugs used regularly during the last 12 months before death
**PD1**	Levodopa/carbidopa, entacapone, ketoprofen, alimemazine tartrate
**PD2**	Levodopa/carbidopa, pramipexole, furosemide, glimepirid
**PD3**	Levodopa/carbidopa, donezepil, prednisolone, simvastatin, zopiclone, methotrexate, folic acid
**PD4**	Levodopa/carbidopa, mirtazapine, bumetanide
**PD5**	Ropinirol, warfarine, rivastigmin
**PD6**	Levodopa/carbidopa/entacapone, pramipexole, irbesartan
**PD7**	Levodopa/carbidopa, pramipexole, metoprolol, isosorbide mononitrate, acetylsalicylic acid, clopidogrel, atorvastatin, ramipril
**PD8**	Levodopa/carbidopa/entacapone, memantine, quatiapine
**PD9**	Levodopa/carbidopa, levodopa/benserazide, mirtazapine, zolpidem,
**PD10**	Levodopa/carbidopa, ropinirol, rasagiline, ezetimibe, metformin, acetylsalicylic acid

Supplementary Table 2. Medication used by individuals with PD. Complete summary of drugs taken systemically and on a regular basis by individuals with PD included in our study during at least the last 12 months before death.

Gene	PD		Controls	
	**n**	***std***	**n**	***std***
**ND1**	35.1	*11.4*	40.0	*13.1*
**ND2**	29.5	*11.7*	33.3	*10.5*
**rRNA**	24.2	*10.0*	25.8	*8.9*
**tRNA**	36.4	*17.1*	37.7	*15.2*

Supplementary Table 3. Counts of heteroplasmic mtDNA mutations per type of transcript in dopaminergic substantia nigra neurons. PD: Parkinson disease; n: mean number of single nucleotide variants (SNV) per 1000 bp, per dopaminergic substantia nigra neuron; std: standard deviation. In both PD and controls, heteroplasmic mutational burden was lowest in the rRNA and ND2 encoding genes and highest in ND1 and t-RNA encoding regions (ANOVA: $P < 0.0001$; multiple group comparisons done by t-test followed by Bonferroni correction). There was no statistically significant difference of gene-specific mutational burden between PD and controls.
